# The Immunodetection of Non-Falciparum Malaria in Ancient Egyptian Bones (Giza Necropolis)

**DOI:** 10.1155/2018/9058108

**Published:** 2018-07-30

**Authors:** Ghada Darwish AL-Khafif, Rokia El-Banna, Nancy Khattab, Tamer Gad Rashed, Salwa Dahesh

**Affiliations:** ^1^Anthropology and Mummy Conservation Lab., Conservation and Research Centre, Ministry of Antiquities, 11521 Cairo, Egypt; ^2^Biological Anthropology Department, National Research Centre, 12311 Giza, Egypt; ^3^Department of Anthropology, Institute of African Research and Studies, Cairo University, 12613 Giza, Egypt; ^4^Research Institute of Medical Entomology, Ministry of Health, 12619 Giza, Egypt

## Abstract

The detection of falciparum malaria in ancient Egyptian remains had been performed by many authors using several methodologies including the use of rapid diagnostic tests. Through the immunochromatographic analysis of bony specimens from Giza skeletal collection dated to Old Kingdom, we provide first evidence of non-falciparum malaria in Ancient Egypt. The histidine-rich protein-2 (HRP2) specific to* Plasmodium falciparum* was absent in 100% of examined samples, while aldolase, common to the four types of plasmodial pathogens causing human malaria, was detected in 56% of individuals with no significant difference between the two tested social groups: high officials (HO) and workers (W). It is suggested that the main risk factor was the presence of residences near natural and artificial waterways, which allowed prolonged contact between the vector and human host.

## 1. Introduction

Human malaria is an infectious disease that is threatening human health in many parts of the world. Most cases are reported among children under 5 years. Many natural or human-made conditions can increase the chance of malaria transmission. The nutritional deficiencies problems may lead to the complication of the situation, especially among the most susceptible groups: children and pregnant women [1]. The most virulent form of human malaria is caused by* Plasmodium falciparum* that is responsible for the highest rate of mortality, while the diseases caused by* P. vivax*,* P. ovale,* and* P. malariae* are considered benign [2]. Abundance of* Anopheles *species responsible for malaria transmission in a given area is greatly influenced by climatic and environmental conditions. A great behavioral variety among different species is reported [3]. Social and cultural factors affect the biomedical burden of malaria; i.e., the perceptions of the disease in a given society, beliefs, cultural standards, and economic and political circumstances influence the development of the disease, mortality, morbidity, and economic costs [4].

Few studies addressed the issue of malaria diagnosis in archeological human remains. They were carried on ancient Egyptian [5–13] and European remains [14–20]. With the exception of the studies carried by Gowland and Western [20] and Smith [12], all of these studies diagnosed the disease through the detection of biomolecules such as aDNA, antigens, and antibodies. Only five studies tried to diagnose non-falciparum forms of the disease: Biannuci et al., 2008; Pinello, 2008; Fornaciari et al., 2010; Gowland and Western, 2012; and Kendall et al., 2016.

The Giza Plateau contains not only the royal pyramids of the 4^th^ Dynasty but also the tombs of high officials (HO) and workers (W) of the Old Kingdom [21] and Heit el-Ghurab or the workmen city of the 4^th^ Dynasty that comprised an important Nile harbor [22].

The aim of this study is to estimate the prevalence of malaria among Giza community during the Old Kingdom (4^th^-6^th^ Dynasties) using malaria rapid diagnostic tests. Both HO and W were subjected to analysis.

## 2. Materials and Methods

### 2.1. Materials

The Giza bone collection is housed in a magazine in the Giza archeological site. The poor preservation state of many skeletons limited the number of examined individuals to 84 from a parent collection consisting of 305 skeletons. These bones were excavated from the following.

(1) The Western Cemetery: it is a cemetery of HO (4^th^-6^th^ Dynasties) located west of the Great Pyramid. It was constructed to include the remains of the literate individuals who represented the elites such as princes, princesses, architects, scribes, priests, and temple officials [23]. It is argued that some owners of these tombs occupied important administrative positions in Heit el-Ghurab [24]. Skeletons under investigation were excavated by the mission of the Supreme Council of Antiquities (1989–1992) [25]. Only 54 individuals were available for examination.

(2) The Southeast Cemetery: it is the cemetery of W (4^th^-5^th^ Dynasties) located southeast of the Sphinx. Buried individuals are those who worked not only in royal structures but also in the Giza HO tombs [21]. The Supreme Council of Antiquities started to excavate skeletons in 1990 [25]. Only 30 individuals were available for examination.

### 2.2. Methods

The eruption of the third molar was used to ensure that a skeleton is belonging to an adult according to Bass [26]. The poor preservation state of 12 skeletons hindered the estimation of age (Table 1).

84 bone specimens from the 84 selected individuals were used in the current study to extract malarial antigens. Mainly, thoracic and lumbar vertebrae were chosen; however, in case of unavailability of vertebrae other bony elements were used such as ribs, cuboids, patellae, calcanea, and tali.

Antigen extraction was performed in the Research and Conservation Centre, Ministry of Antiquities, according to the method described in the study of Fornaciari et al. [15] with the following exceptions:

- The disinfection of the surface of bone elements before spongy bone extraction was performed using isopropanol swabs instead of UV radiation.
- A small sterile disposable medical blade was used to carefully remove a tiny area of the bone surface instead of using a drill. This aimed to ensure a better control and to avoid destruction of fragile archeological bones. A clean and disinfected obturator of an 11G bone marrow biopsy needle was used to obtain rough powder of spongy bone.
- Manual grinding of spongy bone powder was not performed under a laminar flow cabinet as the errors leading to false-positive or false-negative results can not be attributed to unsterile conditions [27].
- In each vial containing 50 mg of bony powder, 100 *μ*l of sterile physiologic solution (0.9% Na Cl) was added instead of 200 *μ*l to allow doubling the antigen concentration.

Malaria diagnostic test used in this study was ABON™ Plus Malaria* P.f*/*Pan* Rapid Test Device (Whole Blood) manufactured by ABON Biopharm (Hangzhou) Co. Ltd. This test can detect histidine-rich protein-2 (HRP2) specific to* P. falciparum* and aldolase common to all species. It was chosen according to the criteria cited in the report of the World Health Organization [28].

Using the IBM SPSS statistic software (version 22), statistical analysis was performed in the Centre for Static and Statistical Studies and Consultation, Institute of Statistical Studies and Research, Cairo University.

## 3. Results

**Figure 1:**
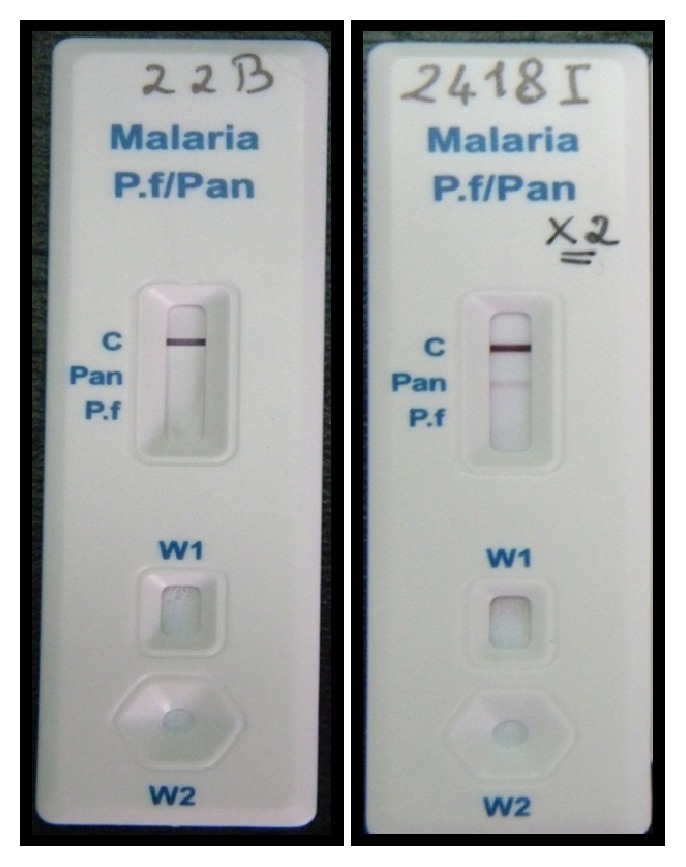
The test on the left is showing a negative result for malaria as only the control line (C) is appearing, while no colored lines are detected at the area of* P. falciparum* (*P.f*.) or that of Pan (the four types of malaria). The test on the right is showing a positive case of non-falciparum malaria as only aldolase band is detected.

**Figure 2:**
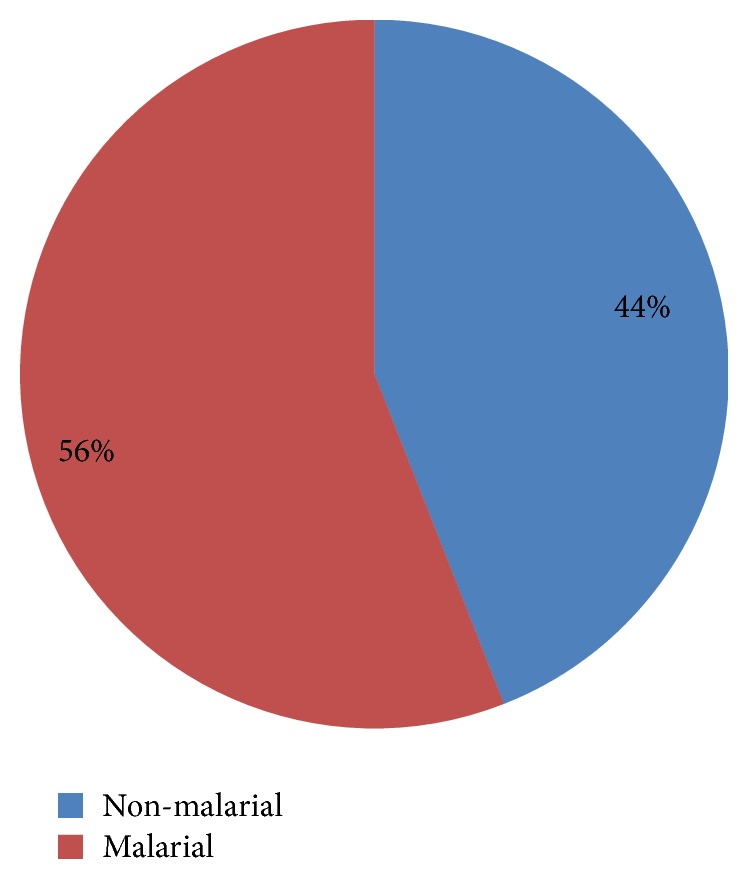
The prevalence of non-falciparum malaria among Giza population.

The presence of malarial antigens was considered equivalent to the infection (Figure 1). Results indicated that 100% of tested individuals were negative for* P. falciparum* antigen, HRP2. The non-falciparum malarial species were detected in 56% of the tested samples, i.e., detection of aldolase in 47 individuals. Thus, no mixed infection with* P. falciparum* was reported (Figure 2).

**Figure 3:**
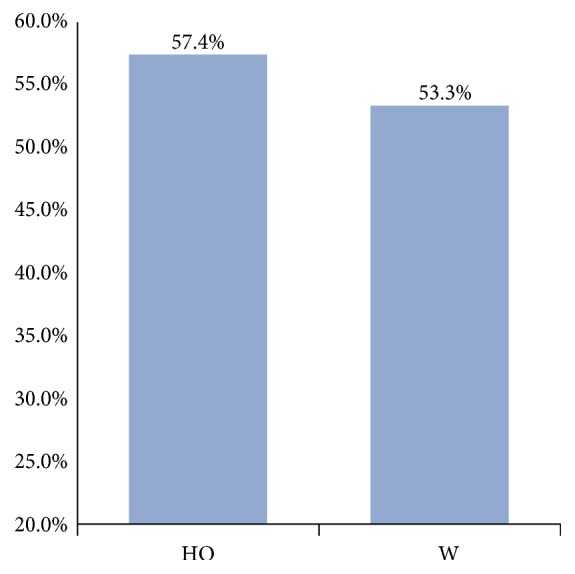
Comparison between prevalences of non-falciparum infection regarding social class.

The prevalence of non-falciparum malaria among HO and W was compared (Table 2 and Figure 3). Pearson's chi-squared test was used to evaluate the significance of difference in malaria prevalence among social ranks. The difference was considered to be statistically significant below the cutoff value 0.05.

Although prevalence was higher in HO, no statistically significant difference between HO and W was reported (p = 0.719).

## 4. Discussion

The immunochromatographic technique was chosen to diagnose malaria in Giza population based on the positive results obtained from previous studies of Miller et al.[9], Cerutti et al. [6], Rabino Massa et al. [11], Bianucci et al. [5], and Fornaciari et al. [15] that detected malarial antigens in ancient human remains, especially that Cerutti et al. [6] and Fornaciari et al. [15] used bony samples; i.e., the stability of antigens over time and the ability to extract it from hard tissues had been proved. Also, although rapid diagnostic tests were invented to diagnose the disease in living patients, it was indicated that it can be used as an effective tool in paleopathological researches. In addition, the use of rapid diagnostic test in the current research was enhanced by its advantages reported by Moody [29]: it is a simple, rapid, economic, and sensitive technique. In contrast, many precautions must be considered in aDNA laboratories to avoid contamination [30]. Also, the aDNA techniques can not be considered economic [31]. In addition, the stability of aDNA is lower than proteinous molecules [32], while the detection of malarial hemozoin and antimalarial immunoglobulins in archeological bones are still virgin fields [33] (Kendall et al., 2010).

The results showed that 56% of the Giza population were infected with non-falciparum malaria, while no infection with falciparum malaria was recorded; i.e., only aldolase was detected. To our knowledge, this is the first time that a non-falciparum antigen is detected in archeological human remains, especially the ancient Egyptian bones. In addition, the previous efforts to detect the biomolecules of* P. vivax* in ancient remains did not meet expected success in spite of presence of historical evidences of malaria endemicity in studied populations,* e.g*., the researches of Pinello [18] and Kendall et al. (2012). Also, no paleopathological studies were performed to detect the presence of the aDNA of* P. ovale* or* P. malariae* in ancient bones until now. The findings indicating infection with non-falciparum malaria match the low mortality rate of subadults in the skeletal collection of Giza: the study of Smith [12] carried out on the commoners of Amarna, Egypt (18^th^ Dynasty), where falciparum malaria was diagnosed indicated that the largest age group of Amarna bone collection was that of children as it reached 42%, while, according to El-Banna [25], the skeletons of subadults comprise only 11% of Giza bone collection.

Many precautions were considered to ensure the authenticity of results. To decrease the chance of having false-positive and false-negative results bones with sun bleaching, salt encrusting, mold growth, or consolidation materials were excluded from analysis. That is why only 84 individuals were chosen for analysis. In addition, a rapid diagnostic test with a high specificity was chosen. According to the kit enclosed instructions, the specificity of ABON Plus Malaria* P.f*/*Pan* Rapid Test Device is over 99% relative to microscopic technique of malaria diagnosis in clinical samples. The World Health Organization Product Testing Programme that is evaluating malaria tests manufactured by many companies around the world recommends using tests with false-positive rate less than 10%. The false-positive rate of ABON Plus Malaria* P.f*/*Pan* Rapid Test Device is 0.4% [28].

As explained by Sallers and Gomzi [19], there are no studies that indicated the mechanism of proteinous biomolecules degradation. Thus, negative results may represent “*an absence of evidence rather than an evidence of absence*” as cited by Bianucci et al. [5] and Bianucci et al. [14]. An argument against the diagenetic effect is the absence of HRP2 accompanied by the presence of aldolase with nearly the same rate in the bones of the two different groups buried in two different cemeteries and dated to the same historical period; i.e., findings reflected the spread of non-falciparum malaria in a specific geographical area during a specific era rather than a variation caused by diagenetic degradation of biomolecules.

Aldolase can persist for days after the clearance of the pathogen from the blood [34]. Thus, its presence in archeological bones can be interpreted as follows: the infected individual died while the pathogen was found in his blood or few days after the cure.

According to the immunochromatographic method used in the current study, it is not possible to determine which species of the three plasmodial pathogens causing non-falciparum malaria was responsible for the disease in Giza population, especially in the absence of textual sources that can be used as indicators for the disease in Giza. However, whatever the species responsible for malaria, the prevalence of the disease must account for the presence of an efficient* Anopheles* vector. As it was demonstrated by Pays [35] and Kenawy [36], there is no evidence indicating which species of mosquito were found in Ancient Egypt, although only two* Anopheles* species have been proved to be the vectors of malaria in Modern Egypt:* An. pharoensis* responsible for the transmission of* P. vivax* in Nile Delta and Valley and* An*. sergentii responsible for* P. falciparum* transmission in Oases [37]. The presence of non-falciparum malaria in ancient Giza may address a question about whether the vector in both ancient and recent eras is the same.

Since it is not possible to determine the species of* Anopheles* that was found in Giza, it is impossible to expect its behavior,* e.g.,* whether the mosquito was exophagic or endophagic. However, it is expected that individuals experienced prolonged contact with the mosquito breeding sites, which allowed the transmission of the disease.

As it was explained by Strouhal [38], the abundance of mosquito breeding sites in Ancient Egypt can be attributed to the annual Nile floodings. This is supported by ancient texts such as in Sabbatani et al. [39]. Also, it is indicated by Noaman and El Quosy [40] that the artificial irrigation system that was practiced over the Dynastic Period involved the retention of the floodwater for about six weeks per year in agricultural lands of Delta and Nile Valley.

According to Love [41], Giza represented a part of the Egyptian capital that was located between the desert and the Nile. According to Lehner [24], a Nile channel was passing near Heit el-Ghurab. Also, this village comprised a major harbor and many waterways.

Since there is no significant difference between the prevalences among HO and W, it is argued that both were subjected to mosquito bites to the same extent due to their residence near the river, waterways and harbor. It is important to note that, according to Hawass [21], a group of workers that occupied the barracks in Heit el-Ghurab worked through a system of rotation; i.e., farmers were brought from all Egyptian nomes every three months to participate in the project of pyramid building. Thus, the present study concludes that some workers buried in the Southeast Cemetery might be infected before coming to Giza due to their residence near the river bank and the application of basin irrigation system in their villages. This may raise the question about the prevalence of the non-falciparum malaria all over Egypt, not only in Giza.

According to Abu-Taleb [42], the social gap inequality increased gradually throughout the Old Kingdom due to the transition from an era of individualism to an era of feudalism. Food quantity and quality in Heit el-Ghurab indicated social inequality [43, 44]. The paleopathological studies of El-Banna [25] and Zaki et al. [45] carried out on the Giza skeletal collection suggested that male W were subjected to a higher degree of nutritional stress than male HO. The findings of the current research indicated that, despite diet stratigraphy, the disease prevalence among W was nearly equal to that among HO. However, this could partially be interpreted as a result of tetracycline ingestion. According to Gaillard et al. [46] tetracycline is used for malaria treatment since 1950. It was argued by Armelagos [47] that the detection of tetracycline in ancient human remains excavated from Dakhla Oasis, Egypt, which is attributed to beer consumption. Throughout the Dynastic Period, beer was available for all social classes and represented an essential part of all meals [48].

The study of Dahesh et al. [49] testing the prevalence of malaria among the recent inhabitants of a village in Fayoum, Egypt, showed that richer individuals showed a lower rate of infection than those belonging to lower rank because of enjoying better sanitation and hygiene and tendency to seek medical care at early stage of infection. The scarcity of evidences concerning cultural adaptation to malaria in Ancient Egypt does not allow an objective analysis of related socioeconomic factors,* e.g.,* although it was suggested that the excavated bed of the Old Kingdom queen Hetepheres might be designed to bear a bednet [38] and the use of mosquito repellent was mentioned in a papyrus [35], there is no evidence about the use of bednets and repellents among HO and W.

Studies about fracture treatment and limb amputation that were carried out on the two social groups by Hussien et al. [50] and Zaki et al. [51] concluded that medical treatment was presented equally to both HO and W. Although this conclusion may contribute to the interpretation of findings of the present work, it is important to note that these studies did not deal with malaria; i.e., the perceptions of the disease and related cultural response cannot be estimated. The research carried out by O'Neill et al. [52] in Gambia manifested the importance of cultural beliefs about malaria: many individuals were not seeking professional medical care because some symptoms of severe malaria such as loss of consciousness were attributed to supernatural entities.

Generally, the absence of significant difference in malaria prevalence regarding social class in Giza reflects the ability of the disease to affect the whole society by the same degree; i.e., the socioeconomic factors did not affect the non-falciparum malaria prevalence in ancient Giza, while it seems that the presence of residences near natural or artificial waterways represented a risk factor.

## 5. Conclusion

Using immunochromatographic analysis of archeological bony samples from Giza Necropolis, we provide first evidence of non-falciparum malaria prevalence in an ancient Egyptian population. This finding may enhance knowledge about the history of the disease that still affects many regions around the world.

Future work will involve studying the relation between the human-made environmental changes involved with urbanism and agricultural practices in Ancient Egypt and the disease prevalence. This will be performed through the comparison of results from Dynastic Period and Predynastic Period. This may include studying the socioeconomic burden of malaria among agricultural societies and hunter-gatherers.

It is recommended to perform aDNA studies on Giza population to detect the species and strains of the non-falciparum pathogen responsible for the disease. The chemical analysis of tetracycline in these archeological bones may extend our knowledge about the relation between dietary behavior and malaria. On a wider level, paleoclimatological researches may add new evidences helping to reconstruct the environmental conditions that permitted the reproduction and the spread of both the vector and the malarial pathogen.

## Figures and Tables

**Table 1:** Number of individuals in each social rank.

	**HO**	**W**	**Total**
**Adults**	45	27	72
**Unknown age**	9	3	12
**Total**	54	30	84

**Table 2:** Malaria prevalence by social class.

**Social class**	**Malarial**	**Non-malarial**	**Total**
HO	31 (57.4%)	23 (42.6%)	54
W	16 (53.3%)	14 (46.7%)	30
**Total**	47 (56.0%)	37 (44.0%)	84

## Data Availability

The photos used to support the findings of this study are included within the article.
